# Alexithymic and autistic traits: Relevance for comorbid depression and social phobia in adults with and without autism spectrum disorder

**DOI:** 10.1177/1362361320936024

**Published:** 2020-07-14

**Authors:** Laura Albantakis, Marie-Luise Brandi, Imme Christina Zillekens, Lara Henco, Leonie Weindel, Hanna Thaler, Lena Schliephake, Bert Timmermans, Leonhard Schilbach

**Affiliations:** 1 Max Planck Institute of Psychiatry, Munich, Germany; 2 International Max Planck Research School for Translational Psychiatry, Munich, Germany; 3 Graduate School of Systemic Neurosciences, Muenich, Germany; 4 University of Muenster, Muenster, Germany; 5 University of Aberdeen, Aberdeen, UK; 6 LVR-Klinikum Duesseldorf/Kliniken der Heinrich-Heine-Universitaet Duesseldorf, Germany; 7 Ludwig-Maximilian-University, Munich, Germany

**Keywords:** adults, alexithymia, autism spectrum disorder, depression, psychiatric comorbidity, social phobia

## Abstract

High alexithymic traits and psychiatric comorbidities such as depression and social phobia are frequently observed among adults with autism spectrum disorder. In this study, we tested whether alexithymic and/or autistic traits are risk factors for depressive and social phobic symptoms in adults with autism spectrum disorder (*n* = 122), patients with social interaction difficulties other than autism (*n* = 62), and neurotypical participants (*n* = 261). Multiple regression analyses of these three groups demonstrated that both traits explained considerable variance of depressive and social phobic symptoms. In adults with autism spectrum disorder, alexithymic traits were predictive of depressive symptoms, while autistic traits predicted social phobic symptoms. In patients with social interaction difficulties other than autism, alexithymic and autistic traits were identified as predictors of social phobic symptoms, while no variable predicted depressive symptoms. In neurotypicals, both alexithymic and autistic traits were predictive of depressive and social phobic symptoms. Our results, therefore, highlight the importance of assessing both alexithymic and autistic traits in patients with and without autism spectrum disorder for identifying comorbid psychopathology. Depending on the underlying core symptomatology, alexithymic and/or autistic traits increase the risk of depressive and social phobic symptoms calling for therapeutic strategies to prevent or at least reduce comorbid psychopathology.

**Lay abstract:**

Adults with autism often develop mental health problems such as depression and social phobia. The reasons for this are still unclear. Many studies found that alexithymia plays an important role in mental health problems like depression. People with alexithymia have difficulties identifying and describing their emotions. Almost every second person with autism has alexithymia. Therefore, we explored in this study whether alexithymia is linked to worse mental health in autistic people. We looked at two common diagnoses, depression and social phobia. We found that alexithymia increased symptoms of depression, while autistic traits increased symptoms of social phobia. Our results suggest that alexithymia and autistic traits can increase the risk of mental health problems. An early assessment could help prevent mental health problems and improve quality of life.

## Introduction

Autism spectrum disorder (ASD) is a neurodevelopmental disorder with core deficits in social communication and social interaction aside from restricted, repetitive patterns of behavior, interests, or activities according to the *Diagnostic and Statistical Manual of Mental Disorders* (5th ed.; *DSM-5*; American Psychiatric Association, 2013) criteria. In addition to the underlying core symptoms of ASD, autistic individuals often experience comorbid mental health problems. Worldwide, depression and social phobia are thought to occur in up to 50% of cases with ASD (Albantakis et al., 2018; Hedley et al., 2018; Maddox & White, 2015; Spain et al., 2016). Moreover, approximately 50% of autistic patients display clinically significant levels of alexithymia (Hill et al., 2004; Kinnaird et al., 2019; Milosavljevic et al., 2016; Morie et al., 2019). This raises the need to enhance our understanding of alexithymia in ASD and its contribution to comorbid mental health problems.

Alexithymia is a subclinical condition in which affected individuals have difficulties identifying and describing their own emotions (Taylor, 2000) as well as the emotions in others, for example, through the interpretation of facial expressions, which is central to the diagnosis of ASD (Cook et al., 2013; Starita et al., 2018). In fact, some researchers have even suggested that emotional impairments seen in ASD are due to concurrent alexithymia rather than representing a genuine feature of autism (Bird & Cook, 2013; Trevisan et al., 2016).

Thus, it is possible that emotion recognition and regulation difficulties of comorbid psychopathology seen in patients with ASD (Aldao et al., 2010; McLaughlin & Nolen-Hoeksema, 2011; Weissman et al., 2019) are associated with alexithymia (Bilotta et al., 2016; Laloyaux et al., 2015). In addition, high levels of autistic traits have similarly been associated with altered emotion processing (Corden et al., 2008; Samson et al., 2012).

**Figure 1.**
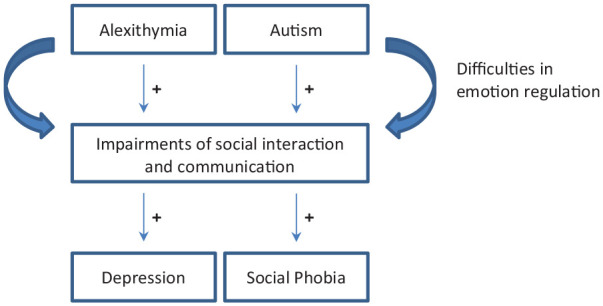
Model depicting the positive associations of alexithymic and autistic traits on depression and social phobia through difficulties in emotion regulation.

Indeed, maladaptive emotion regulation strategies like suppression and avoidance are typical for people with alexithymic, but also with autistic traits (e.g. Bilotta et al., 2016; Jahromi et al., 2012; Laloyaux et al., 2015; Samson et al., 2015). They can negatively affect social interactions resulting in a lack of social support and potentially mood and anxiety disorders (Figure 1; Hofmann, 2014; Marroquín, 2011; Williams et al., 2018). Importantly, previous studies have shown that alexithymic traits (Hemming et al., 2019; Leweke et al., 2012; Pandey et al., 2011) and autistic traits (Fietz et al., 2018; Lundström et al., 2011; Morie et al., 2019) may both act as a vulnerability factor for mental illness, notably depressive and anxiety disorders. This is highly relevant because (comorbid) mental health problems, in particular depressive disorders, have been identified as a major risk factor for suicidal ideation and suicide attempts in autistic children (Mayes et al., 2013) and suicide represents the second leading cause of death in adult individuals with ASD (Hirvikoski et al., 2016). These dramatic findings highlight the need to improve our understanding of alexithymic and autistic traits in comorbid psychopathology of ASD.

### Study aims

Our study aimed to investigate whether the degree of autistic and alexithymic traits can explain the co-occurrence of depressive and social phobic symptoms. Since autistic and alexithymic traits are dimensional constructs and present in both clinical and non-clinical populations (Austin, 2005; Fietz et al., 2018; Franz et al., 2008; Ingersoll & Wainer, 2014; Kamp-Becker et al., 2010; Ruzich et al., 2015; Salminen et al., 1999), we extended the investigation beyond ASD to include other patients with social interaction difficulties other than autism and the general population. This approach was chosen in concordance with a dimensional and transdiagnostic approach to mental health, as for instance set out in the National Institute of Mental Health (NIMH) Research Domain Criteria (RDoC) framework (Insel et al., 2010), which had been implemented in a specialized “Clinic and Day Clinic for Disorders of Social Interaction” at the Max Planck Institute of Psychiatry. Consequently, we included patients with a confirmed diagnosis of ASD, patients who showed social interaction difficulties, but did not receive a formal diagnosis of ASD as well as neurotypicals. In light of the dimensional character of alexithymic and autistic traits and their existence in both clinical and non-clinical populations, we investigated the degree to which both traits contribute to depressive and social phobic symptoms irrespective of the underlying core condition.

## Methods

### Participants

**Figure 2.**
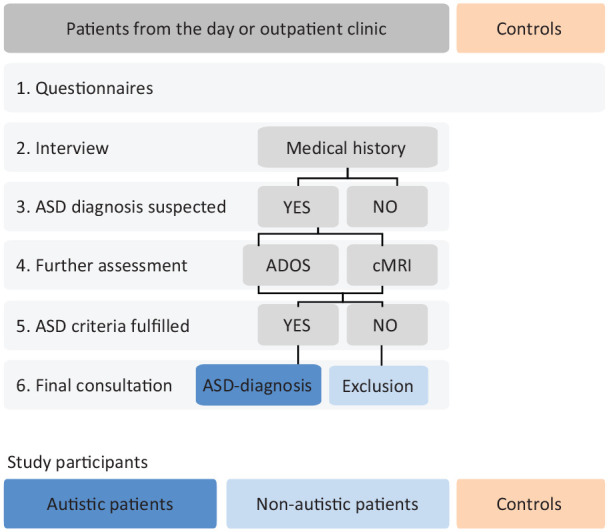
Diagnostic procedure for study participants. ADOS: Autism Diagnostic Observation Schedule; cMRI: cranial magnetic resonance imaging; ASD: autism spectrum disorder.

The study included three groups of participants: participants with a confirmed diagnosis of ASD; participants with social interaction difficulties, but no diagnosis of ASD; and neurotypicals without any social communicative impairments. Participants with a confirmed or an excluded diagnosis of ASD were all patients admitted to the “Outpatient and Day Clinic for Disorders of Social Interaction” at the Max Planck Institute of Psychiatry in Munich, Germany, from April 2015 to January 2018 (Table 1). They had been referred to the clinic for ASD evaluation. All patients received a diagnostic assessment for ASD according to the national autism guidelines (Arbeitsgemeinschaft der Wissenschaftlichen Medizinischen Fachgesellschaften [AWMF], 2015). (Figure 2). Depending on the results of the diagnostic evaluation, patients were divided into two groups: Individuals of the first group fully met the *DSM-5* criteria of ASD (*n* = 122) and received the diagnosis of ASD. They are referred to as “ASD group.” Since patients of the ASD group did not have any intellectual impairment, they were regarded as patients with high-functioning autism. Individuals of the second group showed significant impairments of their social communicative skills, but did not fulfill the diagnostic criteria of ASD according to *DSM-5* (*n* = 62). For example, patients presented with social interaction difficulties but did not give any indication for repetitive and restrictive patterns of behavior, interests, or activities. They are referred to as “non-ASD group.” We also included a third group of neurotypical participants (*n* = 261) defined as adults without any history of psychiatric or neurological impairments. Subjects of this group had taken part in research projects within the independent Max Planck Research Group for “Social Neuroscience” at the Max Planck Institute of Psychiatry in the past. All study participants (*N* = 445) provided written informed consent. Ethical approval was granted by the Ethics Committee of the Ludwig-Maximilian-University, Munich. All procedures were performed in accordance with the Declaration of Helsinki.

**Table 1.** Characteristics of participants.

Variables	ASD	non-ASD	NT	*F*	*p* value	Difference
*N*	122	62	261	–	–	–
Sex (m/f)	83/39	37/25	120/141	–	–	–
Alexithymia (yes/no)	68/54	30/32	11/250	–	–	–
Mean ADOS (*SD*)	7.01 (3.17)^a^	3.95 (2.97)^b^	–	–	<0.001	ASD > non-ASD^c^
Mean age in years (*SD*)	33.46 (10.40)	35.15 (12.62)	26.41 (7.80)	36.47	<0.001	non-ASD > ASD > NT
Mean AQ (*SD*)	36.25 (8.16)	32.29 (9.39)	15.08 (5.60)	441.18	<0.001	ASD > non-ASD > NT
Mean TAS-20 (*SD*)	62.18 (10.90)	59.36 (10.72)	44.36 (9.71)	149.45	<0.001	ASD > non-ASD > NT
Mean BDI-II (*SD*)	17.30 (11.59)	21.92 (10.54)	5.40 (5.33)	148.41	<0.001	non-ASD > ASD > NT
Mean LSAS (*SD*)	77.28 (26.81)	70.55 (29.17)	31.26 (18.36)	203.37	<0.001	ASD > non-ASD > NT

ASD: patients with autism spectrum disorder; non-ASD: patients with social interaction difficulties, but no diagnosis of ASD; NT: typically developing group; TAS-20: Toronto Alexithymia Scale-20 (scale: 20–100); Alexithymia: TAS-20 scores ⩾ 61; AQ: Autism-Spectrum Quotient (scale: 0–50); BDI-II: Beck Depression Inventory-II (scale: 0–63); LSAS: Liebowitz Social Anxiety Scale (scale: 0–144); *SD*: standard deviation; ADOS: Autism Diagnostic Observation Schedule; ANOVAs: analyses of variance.

ANOVAs with contrasts were calculated to determine group differences. Results are based on 1000 bootstrap samples.

a Available information for *n* = 102.

b Available information for *n* = 41.

c Unpaired *t* test: *t*(141) = −5.31, *p* < 0.001.

### Clinical data

Medical and psychosocial histories from all patients (ASD and non-ASD group) were assessed in interviews conducted by a psychologist or psychiatrist experienced in diagnosing ASD (Supplementary Table S1 for psychiatric comorbidities). As part of the regular diagnostic process for ASD, patients (ASD and non-ASD group) were tested using the “Autism Diagnostic Observation Schedule-2” (ADOS-2; Hus & Lord, 2014) (Table 1).

### Measures

Participants of all three groups (ASD, non-ASD, and neurotypicals) were asked to fill out the same set of psychometric questionnaires. To evaluate autistic traits, the “Autism-Spectrum Quotient (AQ)” (Baron-Cohen et al., 2001) was used. The “Toronto Alexithymia Scale” with 20 items (TAS-20) (Bagby et al., 1994) reflected the level of alexithymia in individuals, whereas the “Liebowitz Social Anxiety Scale (LSAS)” (Fresco et al., 2001) and “Beck Depression Inventory-II (BDI-II)” (Beck et al., 1996) provided information about social phobic and depressive symptoms, respectively. Only data sets with less than 10% of missing data per instrument were included (Table 1).

### Analyses

Data processing and statistical analyses were performed in MATLAB (R2010a, The MathWorks, Inc., Natick, MA, USA) and IBM SPSS 25.0 including the bootstrapping tool (IBM Corp., 2017). Tests of normality revealed that the relevant measures (age, AQ, BDI-II, TAS-20, LSAS) were not normally distributed neither in the total sample (including all three groups) nor in each subgroup (Kolmogorov–Smirnov tests, all *p* values < 0.05). Data transformation failed to improve skewness. Therefore, correlational, logistic, and multinomial regression analyses were performed with 1000 resamples bootstrapping to provide more robust statistics (Field, 2018).

### Models

**Figure 3.**
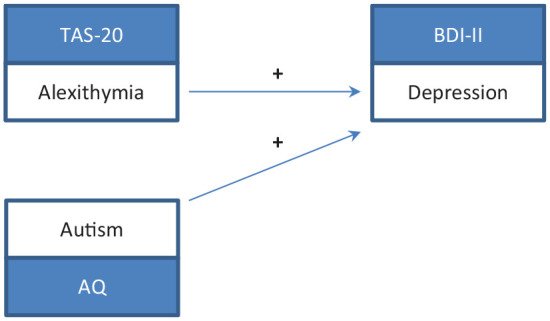
Model depicting the positive associations of alexithymic and autistic traits on depression. TAS-20: Toronto Alexithymia Scale-20; BDI-II: Beck Depression Inventory-II; AQ: Autism-Spectrum Quotient.

**Figure 4.**
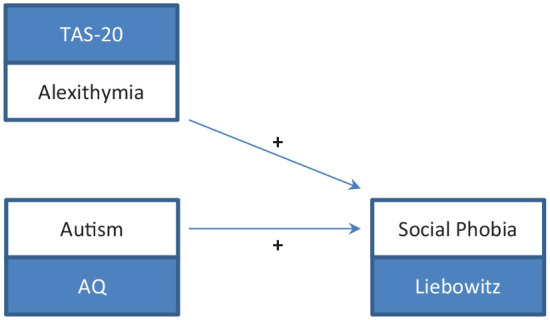
Model depicting the positive associations of alexithymic and autistic traits on social phobia. TAS-20: Toronto Alexithymia Scale-20; AQ: Autism-Spectrum Quotient.

We generated two models for testing our hypotheses and performed the analyses for each group separately. In the first model (Figure 3), we tested whether autistic, alexithymic, or both traits explained variance of depressive symptoms. In the second model (Figure 4), we tested whether autistic, alexithymic, or both traits explained variance of social phobic symptoms. For each model, we performed a three-step, forced entry hierarchical regression analysis according to the procedures established by Cook and colleagues (2013), with depressive symptoms as dependent variable in model 1 and social phobic symptoms in model 2. To explain the approach in short: In a first step, predictors of no interest (in our case, age and sex) were included in the model. In the following step, predictors of interest (in our case, alexithymic and autistic traits) were added to the model, first alexithymic, then autistic traits. By doing this, we could observe how much additional variance was explained by each predictor of interest. Multicollinearity of predictor variables was checked for by computing the variance inflation factor (VIF) and the tolerance statistic for each analysis. Values of VIF and of tolerance were below cut-off criteria in all analyses, suggesting that multicollinearity was negligible. Although tests did not indicate significant multicollinearity, moderate correlations between TAS-20 and AQ scores were found in each group (see Supplementary Table S2). Since this might bias the estimated roles of predictors in the model, we computed two further regressions changing the order of entries of alexithymic and autistic traits in the analyses. Thus, our complete computational pathway can be summarized as follows: In the first step (1), age and sex were entered in the model. Second (2 A), alexithymic traits were added. Finally (3 A), autistic traits were included. Then the order of entries was changed. So autistic traits were added second (2 B), and alexithymic traits last (3 B).

### Correlations between alexithymic and autistic traits

To identify associations among the variables, simple uncorrected correlation analyses were run for each group (Supplementary Table S2).

## Results

### Characteristics of participants

Patients with a confirmed diagnosis of ASD had significantly higher ADOS scores than patients with no ASD diagnosis (Table 1). Alexithymia, defined as TAS-20 scores ⩾ 61 according to Bagby and colleagues (1994), was found in 55.7% of autistic and 48.4% of non-autistic patients, while only 4.2% of neurotypicals had alexithymia. Patients with ASD and non-ASD showed similar rates of psychiatric comorbidities (Supplementary Table S1).

### Alexithymic and autistic traits predicting depressive symptoms

To address our hypothesis that variance in depressive symptoms can be explained by both autistic and alexithymic traits, we observed for each group separately the stepwise changes of variance induced by entering alexithymic and autistic traits as predictors in the models (Table 2). The results are described in detail for the ASD group, while the results for the non-ASD and neurotypical groups are summarized in the text (see Table 2 and Supplementary Tables S3a and b for further details).

**Table 2.** Three-step hierarchical regression models for depression.

Groups	Steps	Predictors	*R*^2^ (%)	Δ *R*^2^ (%)	*F* change	Sig. *F* change
ASD	1	Age, Sex	3.2	3.2	*F*(2, 119) = 1.96	0.142
	2 A	Age, Sex, TAS-20	16.4	13.2	*F*(1, 118) = 18.55	<0.001***
	3 A	Age, Sex, TAS-20, AQ	16.4	0	*F*(1, 117) = 0.05	0.820
	2 B	Age, Sex, AQ	8.3	5.1	*F*(1, 118) = 6.47	0.012*
	3 B	Age, Sex, AQ, TAS-20	16.4	8.1	*F*(1, 117) = 11.41	0.001**
Non-ASD	1	Age, Sex	0.2	0.2	*F*(2, 59) = 0.06	0.945
	2 A	Age, Sex, TAS-20	4.3	4.1	*F*(1, 58) = 2.51	0.119
	3 A	Age, Sex, TAS-20, AQ	4.6	0.3	*F*(1, 57) = 0.16	0.689
	2 B	Age, Sex, AQ	2.1	1.9	*F*(1, 58) = 1.14	0.291
	3 B	Age, Sex, AQ, TAS-20	4.6	2.5	*F*(1, 57) = 1.49	0.228
NT	1	Age, Sex	1.3	1.3	*F*(2, 258) = 1.71	0.182
	2 A	Age, Sex, TAS-20	14.5	13.2	*F*(1, 257) = 39.5	<0.001***
	3 A	Age, Sex, TAS-20, AQ	21.1	6.6	*F*(1, 256) = 21.47	<0.001***
	2 B	Age, Sex, AQ	15.8	14.5	*F*(1, 257) = 44.13	<0.001***
	3 B	Age, Sex, AQ, TAS-20	21.1	5.3	*F*(1, 256) = 17.23	<0.001***

ASD: patients with autism spectrum disorder; TAS-20: Toronto Alexithymia Scale-20 (scale: 20–100) measuring alexithymic traits; AQ: Autism-Spectrum Quotient (scale: 0–50) measuring autistic traits; non-ASD: patients with social interaction difficulties, but no diagnosis of ASD; NT: typically developing group.

Results are based on 1000 bootstrap samples.

* *p* < 0.05. **p < 0.01. ***p < 0.001.

In the ASD group, entering alexithymic traits into the model (step 2 A) increased *R*^2^ by 13.2% with alexithymic traits as the significant predictor of depressive symptoms (*β* = 0.40, *p* = 0.001). Including autistic traits last (step 3 A) increased *R*^2^ by 0%. The final model, however, remained significant (*F*(4, 117) = 5.74, *p* < 0.001) with alexithymic traits as the only significant predictor of depressive symptoms (*β* = 0.38, *p* = 0.003). AQ and TAS-20 scores were moderately correlated, *r* = 0.61, *p* < 0.001. Thus, for reasons mentioned above, we changed the order of entries and re-ran the analyses. Entering autistic before alexithymic traits into the model (step 2 B) increased *R*^2^ by 5.1% with autistic traits as the significant predictor of depressive symptoms (*β* = 0.34, *p* = 0.012). Including alexithymic traits last (step 3 B) increased *R*^2^ by additional 8.1% with alexithymic traits as the only significant predictor of depressive symptoms (*β* = 0.38, *p* = 0.001). These results indicate that autistic traits only explained variance in depressive symptoms in the absence of alexithymic traits (step 2 B), while alexithymic traits predicted depressive symptoms irrespective of the inclusion of autistic traits in the models (step 2 A and 3 B).

In the non-ASD patient group, none of the models produced a statistically significant effect. Neither alexithymic nor autistic traits were significant predictors of depressive symptoms.

In the neurotypical group, both alexithymic and autistic traits explained additional variance in depressive symptoms. Alexithymic (*β* = 0.14, *p* = 0.001) and autistic traits (*β* = 0.27, *p* = 0.001) were both significant predictors of depressive symptoms.

Taken together, analyses in the ASD group revealed that autistic traits only predicted depressive symptoms in the absence of alexithymic traits, while alexithymic traits predicted depressive symptoms irrespective of autistic traits in the models. In the non-ASD group, neither alexithymic nor autistic traits were a significant predictor of depressive symptoms, while in the neurotypical group, both alexithymic and autistic traits were significant predictors of depressive symptoms irrespective of the other variable.

### Alexithymic and autistic traits predicting social phobic symptoms

To investigate the relationship of autistic and alexithymic traits on social phobic symptoms, we observed for each group separately the stepwise changes of variance induced by entering alexithymic and autistic traits as predictors in the models (Table 3). The results are described in detail for the ASD group, while the results for the non-ASD and neurotypical groups are summarized in the text (see Table 3 and Supplementary Tables S4a and b for further details).

**Table 3.** Three-step hierarchical regression models for social phobia.

Groups	Steps	Predictors	*R*^2^ (%)	Δ *R*^2^ (%)	*F* change	Sig. *F* change
ASD	1	Age, Sex	10	10	*F*(2, 119) = 6.60	0.002**
	2 A	Age, Sex, TAS-20	21.8	11.8	*F*(1, 118) = 17.78	<0.001***
	3 A	Age, Sex, TAS-20, AQ	32.1	10.3	*F*(1, 117) = 17.72	<0.001***
	2 B	Age, Sex, AQ	31.1	21.1	*F*(1, 118) = 36.23	<0.001***
	3 B	Age, Sex, AQ, TAS-20	32.1	1.0	*F*(1, 117) = 1.60	0.208
Non-ASD	1	Age, Sex	9.6	9.6	*F*(2, 59) = 3.12	0.052
	2 A	Age, Sex, TAS-20	39.6	30.1	*F*(1, 58) = 28.89	<0.001***
	3 A	Age, Sex, TAS-20, AQ	43.2	3.6	*F*(1, 57) = 3.62	0.062
	2 B	Age, Sex, AQ	27.0	17.4	*F*(1, 58) = 13.87	<0.001***
	3 B	Age, Sex, AQ, TAS-20	43.2	16.2	*F*(1, 57) = 16.30	<0.001***
NT	1	Age, Sex	2.5	2.5	*F*(2, 258) = 3.30	0.039*
	2 A	Age, Sex, TAS-20	19.7	17.2	*F*(1, 257) = 55.17	<0.001***
	3 A	Age, Sex, TAS-20, AQ	25.9	6.2	*F*(1, 256) = 21.45	<0.001***
	2 B	Age, Sex, AQ	18.0	15.5	*F*(1, 257) = 48.57	<0.001***
	3 B	Age, Sex, AQ, TAS-20	25.9	7.9	*F*(1, 256) = 27.45	<0.001***

ASD: patients with autism spectrum disorder; TAS-20: Toronto Alexithymia Scale-20 (scale: 20–100) measuring alexithymic traits; AQ: Autism-Spectrum Quotient (scale: 0–50) measuring autistic traits; non-ASD: patients with social interaction difficulties, but no diagnosis of ASD; NT: typically developing group.

Results are based on 1000 bootstrap samples.

* *p* < 0.05. **p < 0.01. ***p < 0.001.

In the ASD group, entering alexithymic traits into the model (step 2 A) increased *R*^2^ by 11.8% with sex (*β* = 11.40, *p* = 0.011) and alexithymic traits (*β* = 0.87, *p* = 0.001) as significant predictors of social phobic symptoms. Including AQ last (step 3 A) increased *R*^2^ by additional 10.3% with autistic traits (*β* = 1.39, *p* = 0.001) as the only significant predictor of social phobic symptoms in the final model. AQ and TAS-20 scores moderately correlated, *r* = 0.61, *p* < 0.001. Thus, for reasons mentioned above, we changed the order of entries and re-ran the analyses. Entering autistic before alexithymic traits into the model (step 2 B) increased *R*^2^ by 21.1% with autistic traits (*β* = 1.63, *p* = 0.001) as the significant predictor of social phobic symptoms (*β* = 1.63, *p* = 0.001). Including alexithymic traits last (step 3 B) increased *R*^2^ by additional 1.0% with autistic traits (*β* = 1.39, *p* = 0.001) as the only significant predictor of social phobic symptoms in the final model, *F*(4, 117) = 13.80, *p* < 0.001. These results indicate that alexithymic traits only explained variance in social phobic symptoms in the absence of autistic traits (step 2 A), while autistic traits predicted social phobic symptoms irrespective of the inclusion of alexithymic traits in the models (step 2 B and 3 A).

In the non-ASD patient and in the neurotypical groups, both alexithymic and autistic traits explained additional variance in social phobic symptoms. Alexithymic (non-ASD: *β* = 1.24, *p* = 0.001, neurotypical: *β* = 0.59, *p* = 0.001) and autistic (non-ASD: *β* = 0.68, *p* = 0.032; neurotypical: *β* = 0.90, *p* = 0.001) traits were significant predictors of social phobic symptoms in the final model.

Taken together, analyses in the ASD group revealed that alexithymic traits only predicted social phobic symptoms in the absence of autistic traits, while autistic traits predicted social phobic symptoms irrespective of alexithymic traits in the models. In the non-ASD and neurotypical groups, both alexithymic and autistic traits were significant predictors of social phobic symptoms irrespective of the other variable.

## Discussion

The present study examined the association of autistic and alexithymic traits with depressive and social phobic symptoms in adults with a confirmed diagnosis of ASD, patients with social interaction difficulties but no diagnosis of ASD, and neurotypical participants. Analyses were performed in these three groups separately to investigate the impact of the underlying core condition on the contribution of alexithymic and autistic traits on depressive and social phobic symptoms.

We observed a high prevalence of alexithymia in the ASD group, which is in line with previous reports of alexithymic traits in autism (Berthoz & Hill, 2005; Kinnaird et al., 2019; Milosavljevic et al., 2016; Morie et al., 2019). Furthermore, the prevalence of depression and social phobia in this group is consistent with other studies about autism in adulthood (Albantakis et al., 2018; Hedley et al., 2018; Maddox & White, 2015; Spain et al., 2016). Thus, our ASD group realistically meets the characteristics of autistic adults including comorbid psychopathology. The prevalence of alexithymia in the non-ASD patient group is also consistent with previous findings in non-autistic patients with depressive and anxiety disorders (Cox et al., 1995; Leweke et al., 2012). The prevalence of alexithymia in the neurotypical group was in line with a recent finding for the general population (Fietz et al., 2018). Importantly, patients from the ASD and non-ASD group in our study showed similar rates of comorbidities, leaving the underlying core condition as the main difference.

The model-based analyses that used depressive symptomatology as the dependent variable revealed that in autistic patients, depression scores were predicted by alexithymic traits, but not by autistic traits. This is in line with results of a study with 68 autistic individuals, in which depression was associated with alexithymia, but not autistic symptoms (Morie et al., 2019). Our study, importantly, replicates this previous finding in a substantially bigger group of patients (122 autistic adults). In addition, autism diagnoses in our study were confirmed using the highest possible assessment standards, whereas Morie et al. (2019) relied on self-reported diagnoses. Furthermore, we found that autistic traits were predictors of depressive symptoms before alexithymic traits were entered in the model. Once alexithymic traits were added, the effect of autistic traits on depressive symptoms did not reach significance anymore. This observation is relevant for future studies highlighting the importance of including alexithymia as a control variable to avoid misinterpretation and a potentially false attribution to ASD. Since most current research on alexithymia suggests that it is a stable personality trait rather than a state-related condition (Hemming et al., 2019), our findings are in favor of an assessment of alexithymic traits in patients with ASD to identify and prevent the risk of a subsequent depressive disorder.

Contrary to our expectations, the analysis of the non-ASD patient group showed that neither alexithymic nor autistic traits were identified as significant predictors of depressive symptoms. This is surprising, given that the non-autistic patients suffered from social interaction difficulties and scored highest on mean BDI-II values. Also, previous studies have shown that alexithymia tends to co-occur with depression (Hemming et al., 2019; Leweke et al., 2012). A possible explanation for not having found a link between alexithymia and depression in the non-ASD patient group may be attributed to a large heterogeneity of psychopathology in this group, which also constitutes an important limitation of our characterization of this subgroup. Also, the non-ASD group was the smallest in number of the three subgroups and therefore underpowered to demonstrate significant effects with any of the variables included.

In the neurotypical group, alexithymic and autistic traits independently accounted for a significant amount of variance in depressive symptoms. These findings are again similar to the results by Fietz and colleagues (2018) who found a small to moderate effect for alexithymic and a moderate effect for autistic traits in predicting depression in the general population.

Regarding social phobic symptoms, we conversely found autistic traits to be significant predictors of social phobic symptoms in the ASD patient group. Our findings thus indicate that in contrast to depressive symptoms, autistic rather than alexithymic traits were predictive of social phobic symptoms in patients with ASD. Again, our results are consistent with results by Morie et al. (2019) who did not observe an association of anxiety and alexithymia. In the non-ASD patient and neurotypical groups, we found that alexithymic and autistic traits were both significant predictors of social phobic symptoms. Results in the neurotypical group stand in contrast to the findings by Fietz and colleagues (2018) who found only alexithymic, but not autistic traits, to be significantly correlated with anxiety-related symptoms. Despite similar sample sizes, the comparability to our study is limited due to the use of different psychometric instruments. Fietz and colleagues (2018) focused on general aspects of anxiety rather than social phobia when examining the interaction of autistic and alexithymic traits.

Taken together, both alexithymic and autistic traits accounted for depressive and social phobic symptoms in the neurotypical group. Furthermore, both traits predicted social phobic symptoms in the non-ASD patient group. Importantly, in the ASD group, only alexithymia but not autistic traits predicted levels of depression, while conversely, autism severity explained variance in social phobia. Therefore, our findings extend previous research which has suggested that alexithymia and not levels of autism may play a particularly important role in emotion processing (e.g. emotion recognition and expression) that may lead to the development of affective disorders (Bird et al., 2010; Cook et al., 2013; Shah et al., 2016; Trevisan et al., 2016). A key element in this discussion could be emotion regulation, briefly summarized as a process of identifying emotions, selecting a reaction to the emotion, and applying strategies to regulate the reaction to the identified emotion (Gross, 2015; Morie et al., 2019). High alexithymic traits were found in depression and social phobia, suggesting that alexithymia might be the link to the psychopathology due to maladaptive emotion regulation strategies that have also been observed in these disorders (Panayiotou et al., 2015; Spokas et al., 2009). However, in patients with ASD, patterns of these maladaptive strategies remained after controlling for alexithymia (Samson et al., 2012), indicating that autistic traits may modulate emotion regulation in addition to alexithymia.

Furthermore, individuals with ASD tend to use cognitive reappraisal, for example, taking another mental perspective on a situation to reinterpret its meaning, less often as an emotion regulation strategy (Samson et al., 2012). This could be due to an impaired ability of perspective taking or theory of mind, commonly found in ASD (Boucher, 2012). This hypothesis is supported by findings from a functional magnetic resonance imaging (fMRI) study, which showed that ASD was associated with atypicalities in brain networks associated with theory of mind functions (Bernhardt et al., 2014). In contrast, alexithymia was associated with brain networks subserving affective processes, for example, emotions and empathy, but not with activation of the theory of mind networks. This dissociation between deficits in sociocognitive and socioaffective networks in ASD and alexithymia could also be relevant for comorbid psychopathology. Given that depression is an affective or emotional disorder, it can be assumed that alexithymia is involved in the pathological process of depression. This was supported by our findings in the ASD and neurotypical group but not in the non-ASD group.

Contrary to our results in depression, autistic traits (including deficits in theory of mind) instead of alexithymic traits seem to be more relevant for social phobia in ASD. While patients with social phobia unlike autistic individuals tend to hyper-mentalize (Ballespí et al., 2019; Hezel & McNally, 2014), deficits of perspective taking could indirectly increase the risk of social phobia in ASD due to negative experiences in social interactions. In other words, due to an impaired theory of mind and other deficits in social interactional skills, autistic individuals are at greater risk of receiving negative social feedback or even of being mocked or bullied by others. Thus, while autistic individuals like social phobic patients may tend to avoid these situations, the underlying mechanisms and reasons may differ.

Importantly, our findings may have direct implications for therapeutic interventions and can help to direct future research. For example, reducing alexithymic traits through psychotherapy could indirectly lead to a differential decrease of depressive and social phobic symptoms across the different patient groups. Beneficial therapeutic strategies for treating alexithymia could be, for example, a group-based setting (Ford & Long, 1977; Swiller, 1988), supportive and educational approaches rather than interpretive approaches (Cameron et al., 2014; Freyberger, 1977; Sifneos, 1975) with a focus on increasing emotional intelligence by teaching emotional components (Amani et al., 2013). Such techniques are also part of group-based psychotherapies for adults with high-functioning autism that are currently being developed (Parpart et al., 2018).

### Strengths and limitations

A strength of our study lies in the relatively large number of study participants (ASD: *n* = 122, non-ASD: *n* = 62, neurotypicals: *n* = 261), which increases the statistical power of the analysis and stands out in comparison with previous research done in this field (e.g. Morie et al., 2019). Furthermore, we applied a multi-sample approach to examine the impact of the underlying core condition on the variables of interest because the origin of the samples (clinical or non-clinical data) matters when investigating psychopathology (Aldao et al., 2010). The statistical analyses chosen for this study are, however, limited with regard to identifying the exact directions or order of events underlying the complex interactions of autistic and alexithymic traits with comorbid psychopathology.

As expected, we found high correlations among our variables of interest (see Supplementary Table S2). However, tests performed indicated that multicollinearity was negligible. In addition, we tackled this issue by applying multiple hierarchical regression analyses with separate entries of TAS-20 and AQ scores including changed orders to control for an effect of order of entry in concordance with previous approaches (Cook et al., 2013; Shah et al., 2016; Trevisan et al., 2016).

## Conclusion

Our study demonstrates that alexithymic and autistic traits are associated with an increased risk of depressive and social phobic symptoms, but that the extent of these associations varies based on the diagnostic group. In the ASD group, only alexithymia but not autistic traits predicted levels of depression, while conversely, autism severity explained variance in social phobia. Consequently, the assessment of alexithymia during the diagnostic procedure for autism seems well warranted, as it may facilitate identifying and reducing the risk for a subsequent depressive disorder including suicidality. In this regard, we support the statement by Trevisan and colleagues (2016) that alexithymia is an important, but too often ignored trait associated with ASD. Future research should aim for an investigation of the behavioral and neural mechanisms of social interaction difficulties associated with autistic and alexithymic traits as this might help to further refine current treatment approaches.

## Supplemental Material

Revised_Supplementary – Supplemental material for Alexithymic and autistic traits: Relevance for comorbid depression and social phobia in adults with and without autism spectrum disorder Click here for additional data file. Supplemental material, Revised_Supplementary for Alexithymic and autistic traits: Relevance for comorbid depression and social phobia in adults with and without autism spectrum disorder by Laura Albantakis, Marie-Luise Brandi, Imme Christina Zillekens, Lara Henco, Leonie Weindel, Hanna Thaler, Lena Schliephake, Bert Timmermans and Leonhard Schilbach in Autism
