# Supplementary material for: Alexithymic and autistic traits: Relevance for comorbid depression and social phobia in adults with and without autism spectrum disorder
Source: Autism. 2020 Jul 14;24(8):2046–56. doi: 10.1177/1362361320936024 (PMC7543015; doi:10.1177/1362361320936024)
Supplement: Revised_Supplementary – Supplemental material for Alexithymic and autistic traits: Relevance for comorbid depression and social phobia in adults with and without autism spectrum disorder [file Revised_Supplementary.pdf]

Table S1

Common psychiatric comorbidities in both patient groups

Comorbidities*	ASD	non-ASD
Depression	70 (57.4)	29 (46.8)
Social Phobia	33 (27.0)	16 (25.8)
AD(H)D	12 (9.8)	6 (9.7)
Obsessive compulsive disorder (OCD)	5 (4.1)	5 (8.1)
No comorbidities	34 (27.9)	17 (27.4)

Note. *According to ICD-10 criteria in N (%); N (%) are not cumulating to 100 % due to multi-comorbidity.
ASD: patients with autism spectrum disorder; non-ASD: patients with social interaction difficulties, but no diagnosis of ASD.

Table S2

Correlations among variables of interest

			Age	Sex	AQ	BDI-II	LSAS	TAS-20
ASD	AQ	*r*	.32	.24	1	.28	.54	.61
		p-value	<.001***	.008**		.002**	<.001***	<.001***
		95 % CI	[0.15, 0.48]	[0.08, 0.39]	[1, 1]	[0.11, 0.42]	[0.43, 0.65]	[0.49, 0.72]
	TAS-20	*r*	.22	.11	.61	.39	.40	1
		p-value	.016	.217	<.001***	<.001***	<.001***	
		95 % CI	[0.05, 0.39]	[-0.06, 0.27]	[0.49, 0.72]	[0.24, 0.54]	[0.25, 0.54]	[1, 1]
non-ASD	AQ	*r*	-.08	.21	1	.14	.48	.46
		p-value	.518	.101		.284	<.001***	<.001***
		95 % CI	[-0.36, 0.16]	[-0.05,-0.46]	[1, 1]	[-0.17, 0.41]	[0.31, 0.63]	[0.25, 0.62]
	TAS-20	*r*	.03	.08	.46	.20	.57	1
		p-value	.813	.550	<.001***	.117	<.001***	
		95 % CI	[-0.22, 0.27]	[-0.17, 0.29]	[0.25, 0.62]	[-0.09, 0.46]	[0.39, 0.71]	[1, 1]
NT	AQ	*r*	.05	-.17	1	.36	.36	.39
		p-value	.423	.006		<.001***	<.001***	<.001***
		95 % CI	[-0.07, 0.16]	[-0.29, -0.5]	[1, 1]	[0.23, 0.46]	[0.26, 0.47]	[0.29, 0.49]
	TAS-20	*r*	-.12	-.05	.39	.38	.40	1
		p-value	.061	.394	<.001***	<.001***	<.001***	
		95 % CI	[-0.24, 0.01]	[-0.17, 0.07]	[0.29, 0.49]	[0.26, 0.46]	[0.28, 0.50]	[1, 1]

ASD: patients with autism spectrum disorder; non-ASD: patients with social interaction difficulties, but no diagnosis of ASD; NT: typically developing group; AQ: Autism Quotient (scale: 0-50) measuring autistic traits; TAS-20: Toronto Alexithymia Scale-20 (scale: 20-100) measuring alexithymic traits.

Note. Results are based on 1,000 bootstrap samples.

**p* < .05. ***p* < .01. ****p* < .001.

Table S3a

Predictors of depressive symptoms

Group	Model	Predictor	*b*	Bias	Std. Error	*p*	BCa 95% CI	
							Lower	Upper
ASD	1	Age	.158	0.000	0.101	.124	-0.037	0.360
		Sex	2.364	-0.019	2.267	.295	-1.903	6.783
	2	Age	.072	-0.003	0.097	.471	-0.125	0.259
		Sex	1.540	0.014	2.132	.474	-2.672	5.837
		TAS-20	.397	-0.001	0.096	.001**	0.203	0.591
	3	Age	.066	-0.003	0.100	.514	-0.140	0.261
		Sex	1.441	0.030	2.113	.501	-2.652	5.695
		TAS-20	.382	-0.001	0.113	.003**	0.163	0.611
		AQ	.036	-0.002	0.144	.807	-0.245	0.310
non-ASD	1	Age	-.037	-0.001	0.105	.715	-0.231	0.179
		Sex	-.064	-0.003	2.891	.982	-5.748	5.543
	2	Age	-.044	0.001	0.099	.654	-0.226	0.156
		Sex	-.427	0.143	2.813	.869	-6.018	4.871
		TAS-20	.201	0.006	0.145	.193	-0.069	0.484
	3	Age	-.040	-0.006	0.102	.702	-0.240	0.164
		Sex	-.637	0.274	2.947	.827	-6.341	5.340
		TAS-20	.175	0.009	0.159	.288	-0.110	0.503
		AQ	.067	-0.023	0.191	.719	-0.367	0.386
NT	1	Age	-.018	-0.001	0.036	.591	-0.090	0.054
		Sex	1.169	0.007	0.649	.073	-0.036	2.466
	2	Age	.012	-0.001	0.032	.684	-0.057	0.071
		Sex	1.411	-0.005	0.603	.026*	0.155	2.555
		TAS-20	.201	0.001	0.041	.001**	0.130	0.287
	3	Age	-.005	-0.001	0.030	.881	-0.067	0.051
		Sex	1.843	-0.013	0.652	.008**	0.494	3.078
		TAS-20	.139	0.001	0.038	.001**	0.072	0.216
		AQ	.271	-0.002	0.076	.001**	0.131	0.425

ASD: patients with autism spectrum disorder; non-ASD: patients with social interaction difficulties, but no diagnosis of ASD; NT: typically developing group; AQ: Autism Quotient (scale: 0-50) measuring autistic traits; TAS-20: Toronto Alexithymia Scale-20 (scale: 20-100) measuring alexithymic traits. Note. Results are based on 1,000 bootstrap samples. *$p < .05$. **$p < .01$.

Table S3b

Predictors of depressive symptoms – changed entry order

Group	Model	Predictor	*b*	Bias	Std. Error	*p*	BCa 95% CI	
							Lower	Upper
ASD	1	Age	.158	0.002	0.105	.134	-0.049	0.365
		Sex	2.364	-0.028	2.305	.314	-2.540	6.831
	2	Age	.078	0.002	0.105	.456	-0.119	0.287
		Sex	1.131	-0.038	2.182	.590	-3.281	5.397
		AQ	.344	0.001	0.129	.012*	0.087	0.593
	3	Age	.066	0.001	0.101	.517	-0.129	0.266
		Sex	1.441	-0.016	2.075	.495	-2.795	5.715
		AQ	.036	0.008	0.147	.810	-0.236	0.336
		TAS-20	.382	-0.008	0.118	.001**	0.144	0.617
non-ASD	1	Age	-.037	0.000	0.102	.731	-0.245	0.163
		Sex	-.064	-0.109	2.872	.975	-5.852	5.579
	2	Age	-.030	-0.006	0.103	.771	-0.240	0.169
		Sex	-.676	0.071	2.969	.828	-6.450	5.214
		AQ	.159	-0.022	0.174	.357	-0.244	0.436
	3	Age	-.040	-0.005	0.099	.688	-0.240	0.149
		Sex	-.637	0.220	2.933	.829	-6.051	5.341
		AQ	.067	-0.023	0.195	.720	-0.365	0.410
		TAS-20	.175	-0.001	0.159	.272	-0.130	0.524
NT	1	Age	-.018	-0.002	0.036	.602	-0.090	0.049
		Sex	1.169	0.024	0.662	.071	-0.059	2.544
	2	Age	-.028	-0.001	0.031	.379	-0.089	0.028
		Sex	1.856	0.015	0.682	.005**	0.528	3.227
		AQ	.368	-0.002	0.078	.001**	0.225	0.525
	3	Age	-.005	-0.001	0.030	.864	-0.064	0.054
		Sex	1.843	0.018	0.672	.003**	0.513	3.193
		AQ	.271	-0.003	0.076	.001**	0.130	0.424
		TAS-20	.139	0.001	0.038	.001**	0.067	0.217

ASD: patients with autism spectrum disorder; non-ASD: patients with social interaction difficulties, but no diagnosis of ASD; NT: typically developing group; AQ: Autism Quotient (scale: 0-50) measuring autistic traits; TAS-20: Toronto Alexithymia Scale-20 (scale: 20-100) measuring alexithymic traits.

Note. Results are based on 1,000 bootstrap samples. *p < .05. **p < .01.

Table S4a

Predictors of social phobic symptoms

Group	Model	Predictor	*b*	Bias	Std. Error	*p*	BCa 95% CI	
							Lower	Upper
ASD	1	Age	.494	0.001	0.197	.015*	0.105	0.877
		Sex	13.202	-0.007	4.481	.003**	4.616	22.337
	2	Age	.305	-0.004	0.194	.127	-0.085	0.670
		Sex	11.396	0.028	4.334	.011*	3.357	20.007
		TAS-20	.869	0.000	0.185	.001**	0.492	1.245
	3	Age	.107	-0.005	0.188	.553	-0.290	0.455
		Sex	7.595	0.056	4.059	.073	-0.365	15.625
		TAS-20	.298	9.305E-05	0.243	.227	-0.179	0.782
		AQ	1.392	-0.006	0.313	.001**	0.805	2.017
non-ASD	1	Age	-.218	0.001	0.281	.433	-0.753	0.323
		Sex	16.603	-0.273	7.425	.032*	1.039	30.885
	2	Age	-.272	0.001	0.245	.256	-0.749	0.223
		Sex	13.895	-0.297	6.201	.028*	1.192	24.933
		TAS-20	1.499	-0.006	0.232	.001**	1.049	1.994
	3	Age	-.235	0.004	0.233	.311	-0.702	0.237
		Sex	11.767	-0.341	6.286	.065	-1.331	23.070
		TAS-20	1.235	0.004	0.262	.001**	0.714	1.780
		AQ	.678	0.001	0.301	.030*	0.080	1.255
NT	1	Age	.099	-0.002	0.150	.516	-0.201	0.392
		Sex	5.702	0.073	2.193	.015*	1.551	9.859
	2	Age	.218	0.001	0.137	.108	-0.056	0.490
		Sex	6.653	-0.006	1.983	.001**	2.796	10.403
		TAS-20	.791	0.002	0.119	.001**	0.559	1.046
	3	Age	.163	3.704E-05	0.131	.208	-0.086	0.420
		Sex	8.094	0.041	1.955	.001**	4.170	11.941
		TAS-20	.586	0.001	0.122	.001**	0.354	0.832
		AQ	.903	0.005	0.204	.001**	0.509	1.304

ASD: patients with autism spectrum disorder; non-ASD: patients with social interaction difficulties, but no diagnosis of ASD; NT: typically developing group; AQ: Autism Quotient (scale: 0-50) measuring

autistic traits; TAS-20: Toronto Alexithymia Scale-20 (scale: 20-100) measuring alexithymic traits.
Note. Results are based on 1,000 bootstrap samples. *p < .05. **p < .01.

Table S4b

Predictors of social phobic symptoms - changed entry order

Group	Model	Predictor	*b*	Bias	Std. Error	*p*	BCa 95% CI	
							Lower	Upper
ASD	1	Age	0.494	-0.006	0.184	.013*	0.112	0.856
		Sex	13.202	0.079	4.572	.005**	4.204	21.758
	2	Age	0.116	-0.005	0.178	.506	-0.264	0.442
		Sex	7.353	0.157	4.157	.081	-0.878	15.291
		AQ	1.632	0.006	0.228	.001**	1.211	2.105
	3	Age	0.107	-0.006	0.182	.544	-0.277	0.450
		Sex	7.595	0.149	4.235	.068	-0.937	15.417
		AQ	1.392	0.002	0.306	.001**	0.791	1.984
		TAS-20	0.298	0.002	0.232	.207	-0.164	0.780
non-ASD	1	Age	-0.218	-0.003	0.291	.446	-0.780	0.370
		Sex	16.603	0.104	7.411	.031*	2.724	31.811
	2	Age	-0.163	-0.003	0.252	.526	-0.662	0.345
		Sex	11.495	-0.054	6.880	.094	-1.928	25.665
		AQ	1.330	0.026	0.300	.001**	0.807	1.991
	3	Age	-0.235	-0.003	0.234	.319	-0.677	0.252
		Sex	11.767	-0.201	6.409	.081	-0.873	24.801
		AQ	0.678	0.017	0.301	.032*	0.151	1.331
		TAS-20	1.235	-0.009	0.262	.001**	0.676	1.718
NT	1	Age	0.099	-0.004	0.152	.529	-0.197	0.381
		Sex	5.702	0.033	2.216	.009**	1.382	10.225
	2	Age	0.064	-0.001	0.140	.661	-0.211	0.346
		Sex	8.150	-0.013	2.141	.001**	3.648	12.180
		AQ	1.309	0.000	0.203	.001**	0.926	1.745
	3	Age	0.163	0.000	0.136	.240	-0.094	0.427
		Sex	8.094	-0.037	2.020	.001**	4.116	11.837
		AQ	0.903	-0.002	0.198	.001**	0.515	1.319
		TAS-20	0.586	-0.001	0.115	.001**	0.374	0.819

ASD: patients with autism spectrum disorder; non-ASD: patients with social interaction difficulties, but no diagnosis of ASD; NT: typically developing group; AQ: Autism Quotient (scale: 0-50) measuring autistic traits; TAS-20: Toronto Alexithymia Scale-20 (scale: 20-100) measuring alexithymic traits.
Note. Results are based on 1,000 bootstrap samples. *p < .05. **p < .01.
